# Severe traumatic brain injury and long-term survival: a meta-analysis on life expectancy and mortality trends

**DOI:** 10.1007/s10072-025-08706-6

**Published:** 2026-04-10

**Authors:** Donatella Saviola, Stefania Bruni, Jacopo Bonavita, Alessandro Zadra, Mauro Ciavarella, Giovanni Cannavò, Antonio De Tanti

**Affiliations:** 1 Centro Cardinal Ferrari KOS-Care, Via IV Novembre 21, Fontanellato, Parma, 43018 Italy; 2 Neurorehabilitation Unit, Villa Rosa Hospital, APSS Trento, Italy; 3 Section of Legal Medicine, San Carlo Hospital, Potenza, Italy; 4 Società Scientifica Melchiorre Gioia, Pisa, Italy

**Keywords:** Life expectancy, Severe Traumatic Brain Injury (TBIs), Long-term outcome, Mortality, Head injury, Meta-analysis

## Abstract

**Introduction:**

Traumatic brain injury (TBI) is associated with sustained excess mortality and reduced life expectancy compared with demographically matched populations. Our aim was to clarify the magnitude of this burden and its modifiers.

****Material and methods**:**

A random-effects meta-analysis of longitudinal observational studies published through July 2025 was performed. PubMed/Medline, Cochrane Library, Google Scholar, PEDro, and EMBASE were searched for studies reporting standardized mortality ratios or life-expectancy data.

**Results:**

The overall pooled standardized mortality ratio was 3.19 (95% CI 2.59–3.92; I² = 67.4%), estimated by restricted maximum likelihood with Hartung–Knapp adjustment. Analyses stratified by data-collection era demonstrated a progressive decline in relative mortality from 3.95 (1974–1987) to 2.18 (2004–2019; *p* = 0.002). Injury-severity gradients based on Glasgow Coma Scale yielded ratios of 4.51, 2.84, and 1.67 for severe, moderate, and mild injuries, respectively (*p* < 0.001). Age-specific analyses in contemporary cohorts showed the highest relative risk in middle-aged survivors (SMR 7.18 for ages 35–54) and lower ratios in older groups (SMR 2.41 for ≥ 75 years). Mortality peaked within two years post-injury (SMR 6.34) but remained elevated beyond ten years (SMR 2.87). Meta-regression identified study era, follow-up duration, mean age, proportion of severe cases, and sample size as key modifiers, explaining 84.7% of between-study variance. Weighted mean life-expectancy reduction across eight cohorts was 7.6 years.

**Conclusion:**

These findings quantify long-term survival burden after TBI and support the development of comprehensive, lifelong management strategies.

**Supplementary Information:**

The online version contains supplementary material available at 10.1007/s10072-025-08706-6.

## Introduction

Traumatic brain injury (TBI) remains a leading cause of death and long-term disability worldwide [1], affecting millions annually and imposing a heavy toll on patients, families, and healthcare systems [1–3]. TBI has been a major cause of death in young adults [4], but demographic changes have led to a notable rise in TBI incidence among the elderly [5, 6], largely driven by an increase in fall-related injuries, reflecting an aging population [7–10]. Globally, an estimated 64–74 million new TBI cases occur each year, many resulting in long-term impairments [11, 12]. These epidemiologic data emphasize the impact of TBI: survivors often need prolonged hospitalization, rehabilitation, and community care, placing a heavy strain on families and healthcare resources [1, 13]. While acute care for TBI has improved, it is now recognized that TBI can act as a chronic health condition with persistent consequences [14, 15]. One significant long-term consequence is a reduction in life expectancy among TBI survivors [16, 17]. Multiple cohort studies and registry analyses have shown that individuals who survive moderate or severe TBI have higher all-cause mortality than the general population [18]. Yet, the estimated impact of TBI on long-term survival varies greatly across studies due to variations in study design, methodology implemented, patient selection, and follow-up periods [19]. Additionally, most existing data come from specialized centers or national registries, mainly in North America, which may limit their applicability elsewhere [16]. Crucially, prognostic factors such as age at injury and injury severity have not been systematically examined in prior reviews, and outcomes for older age groups (e.g., 65–74 years and ≥ 75 years) remain poorly understood.

Building on a previously published systematic review [16], this meta-analysis provides a rigorous quantitative synthesis of long-term mortality and life expectancy after TBI, incorporating data through 2025 to address these unresolved questions. The updated literature search identified no new eligible studies beyond those included in the prior review, except for one additional study predating 2023. Unlike the previous work that primarily summarized qualitative evidence on long-term mortality following severe TBI, this analysis advances the field by quantitatively estimating both standardized mortality ratios and life expectancy reductions across heterogeneous cohorts. This meta-analytic framework allows for a robust evaluation of how key factors influence survival outcomes, providing clinicians and policymakers with precise quantitative metrics to inform decision-making. Furthermore, the inclusion of meta-regression analysis sheds light on study-level moderators, representing a significant step forward in defining the sustained impact of severe TBI on survival. Secondary aims encompass exploring sources of heterogeneity, including geographic variation and methodological differences across studies. Collectively, this comprehensive synthesis aims to clarify the magnitude of excess mortality associated with TBI and its broader implications.

Ultimately, elucidating patterns of long-term survival after TBI carries substantial clinical and public health importance. Providing accurate prognostic information supports tailored patient counseling, targeted interventions, and strategic resource allocation. This work deepens our understanding of TBI’s lifelong consequences and underpins evidence-based clinical practices and policies aimed at improving outcomes for survivors.

## Materials and methods

This systematic review and meta-analysis build upon a previously published systematic review [16] with some methodological enhancements. Compared to that initial work, we updated the search strategy by replacing Web of Science with EMBASE as a database source, slightly refining the search terms, and incorporating a quantitative synthesis component. Here, we briefly summarize the methodological approach; for a more detailed description, please refer to the previous systematic review [16]. 

The present study was conducted following the Preferred Reporting Items for Systematic Reviews and Meta-Analyses (PRISMA) guidelines [20, 21]. To identify all relevant studies, a comprehensive and reproducible search was performed across multiple databases from January 1, 2000, to July 31, 2025. Data extraction and statistical analyses were completed prior to this update (May 31, 2025), and the July 31, 2025, search did not yield any new eligible studies. The search focused on studies investigating the relationship between traumatic brain injury (TBI) and long-term outcomes, including life expectancy, mortality, and survival. We systematically searched PubMed, Cochrane Library, Google Scholar, PEDro, and EMBASE. The search strategy combined Medical Subject Headings (MeSH) and free-text terms across key conceptual domains such as exposure (e.g., “severe traumatic brain injury”), long-term outcomes (e.g., “life expectancy,” “mortality”), and follow-up design (e.g., “follow-up studies”). Detailed search strategies for each database, including syntax variations, filters, and MeSH term hierarchies, are provided in the Supplementary Materials (see Supplementary 1). All retrieved records were imported into EndNote for duplicate removal, then screened using Rayyan by three independent reviewers SB, DS, and ADT against predefined inclusion criteria. To reduce publication bias and enhance retrieval sensitivity, reference lists of included studies and relevant reviews were manually screened for additional eligible articles. Only studies published in English and reporting the necessary data were considered.

### Eligibility criteria and study selection

We applied predefined eligibility criteria aligned with the PICO framework, consistent with the objectives of this study. These criteria, as well as the study selection process, were based on those detailed in the previous systematic review [16]. Briefly, included studies were longitudinal observational designs enrolling adults (≥ 16 years) with severe TBI (GCS < 9, AIS ≥ 3, ISS ≥ 16), with relevant outcomes related to long-term mortality and life expectancy. In instances where included studies reported cohorts combining mild, moderate and severe TBI cases, data were extracted following the original severity classifications, and sensitivity analyses were planned to evaluate the potential impact of mixed severity samples on the meta-analytic results.

Exclusion criteria encompassed studies lacking extractable mortality data, non-traumatic injuries, heterogeneous veteran populations, and grey literature. Study selection involved independent screening by three reviewers SB, DS, and ADT using a two-stage process (title/abstract and full-text review), with disagreements resolved by consensus. The process has documented according to PRISMA 2020 guidelines as in the original review.

## Data collection, extraction, and reporting

Data extraction followed a standardized form capturing study characteristics, subject demographics, exposure severity, outcomes (e.g., standardized mortality ratios, life expectancy), follow-up duration, and statistical measures. Data were checked for completeness by three independent authors SB, DS, and ADT [16], with discrepancies resolved by discussion. Details of the full extraction procedure are provided in De Tanti et al., 2024 [16]. 

## Methodological quality and risk bias assessment of literature

The methodological quality and level of evidence of each study included in this meta-analysis were independently assessed by three authors SB, DS, and ADT [16] using two instruments applied in the previous review, the Methodological Index for Non-Randomized Studies (MINORS) [22] and the Oxford Centre for Evidence-Based Medicine 2011 Levels of Evidence (OCEBM) [23], with the addition of the Newcastle-Ottawa Scale (NOS) in this updated analysis [24]. The MINORS tool is suitable for both comparative and non-comparative observational studies, whereas the OCEBM classification provides an overall hierarchy of evidence ranging from Level 1 (highest) to Level 5 (lowest). For a detailed description of the MINORS and OCEBM tools, please refer to the previous work [16]. 

The Newcastle-Ottawa Scale was newly introduced to evaluate cohort and case-control studies. Scoring procedures for this scale are described in Supplementary materials (see *Supplementary* 2).

Discrepancies in scoring were resolved by discussion. The results of these risk of bias evaluations are summarized in Table 2 and were used to guide sensitivity analyses conducted to evaluate the robustness of the pooled effect estimates.

### Data analysis

Statistical analyses were conducted to assess the primary outcomes of long-term mortality and reduced life expectancy following traumatic brain injury. Effect size measures included standardized mortality ratios (SMRs), hazard ratios (HRs), odds ratios (ORs), risk ratios (RRs), mean or median survival times, and years of life lost (YLL), depending on the reporting format of each study.

All pooled estimates were calculated using random-effects models [25]. The restricted maximum likelihood (REML) estimator [26, 27] was applied to estimate between-study variance (τ²), and the Hartung–Knapp adjustment [28, 29] was used for the calculation of confidence intervals.

Meta-analyses were stratified by TBI severity (mild, moderate, severe), age groups, duration of follow-up (< 2, 2–5, 5–10, ≥ 10 years), study era (early: 1974–1987; middle: 1988–2003; recent: 2004–2019), and cause-specific mortality categories (e.g., circulatory, respiratory, external, neoplasms, infectious disease, suicide). Studies using alternative classification criteria for severity (e.g., AIS or ISS) were also analyzed separately. Prediction intervals and sensitivity analyses (fixed-effects, leave-one-out, and influence diagnostics) were conducted to test robustness. Publication bias was evaluated with funnel plots, Egger’s and Begg’s tests, and trim-and-fill methods.

All analyses were performed using the R statistical environment (version 4.2.2) and summary tables, forest plots, and funnel plots were constructed to illustrate findings.

Detailed procedures for data extraction, effect size computation, heterogeneity assessment, and sensitivity analyses are provided in Supplementary Materials (see Supplementary Methods).

## Results

### Study selection

**Fig. 1:**
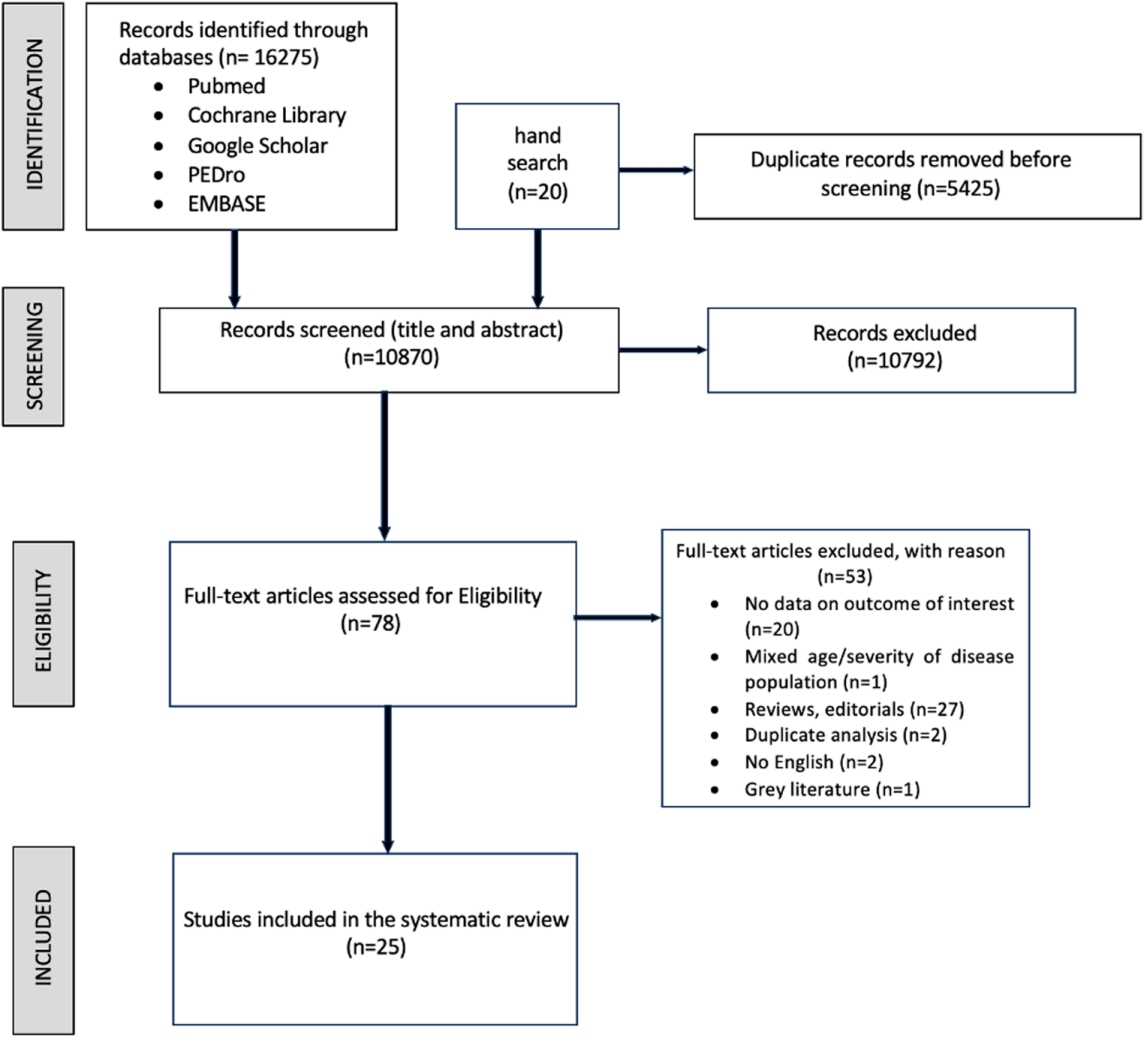
Flow diagram of the literature selection process

A total of 16,275 records were identified through database searches, of which 5425 duplicates were removed. After title and abstract screening, 78 articles were retained for full-text review. Following full-text assessment, 25 studies met all eligibility criteria and were included in the meta-analysis. Studies were mainly excluded for (1) not reporting data on outcomes of interest; (2) reporting data on populations with mixed age and/or disease severity; (3) being reviews or editorials; and (4) reporting duplicate analysis. The study selection process is depicted in the updated PRISMA flow diagram (Fig. 1).

## Characteristics of included studies

The current systematic review with the meta-analysis included 25 studies published between January 1, 2000, and July 31, 2025, encompassing a total of 471,491 individuals diagnosed with TBI. This most recent search identified one eligible study beyond those included in our previous systematic review, even though this study was published before 2023. Studies varied in geographic location, population characteristics, follow-up duration, and TBI severity. Sixteen studies (64%) [30–45] were conducted in the United States, two in Canada (8%) [46, 47], two in Australia (8%) [48, 49], two in Europe (8%) [50, 51], one in the United Kingdom (4%) [52], one in Israel (4%) [53], and one across EU, UK, and Australia (4%) [54]. Study designs included retrospective cohorts (*n* = 15, 60%) [30, 32, 34–39, 41, 43, 44, 46, 48, 49, 52], prospective cohorts (*n* = 7, 28%) [40, 42, 45, 47, 51, 53, 54], population-based cohorts (*n* = 2, 8%) [31, 33], and one cross-sectional study (*n* = 1, 4%) [50]. The median sample size was 1786.5 participants (range: 97–374636). The follow-up duration varied considerably across studies, from 6 months to 27 years. Follow-up periods from 0 to 5 years were reported in 8 studies (32%); [30, 31, 37, 41, 43, 45, 47, 54] follow-ups from 5 to 10 years were reported in six studies (24%) [33, 35, 36, 38, 46, 51], and a follow-up longer than 10 years was reported in ten studies (40%) [32, 34, 39, 40, 42, 44, 48, 49, 53]. The TBI severity assessment utilized the Glasgow Coma Scale in nineteen studies (76%), with severe TBI defined as GCS ≤ 8 in 6 studies [40, 41, 45, 48, 49, 54] and GCS < 13 in 9 studies [30–32, 35, 36, 38, 39, 51, 52]. Four studies [34, 37, 47, 53] included patients with mild to severe TBI, as assessed by GCS. Moderate TBI was defined as GCS 9–12, and mild TBI as GCS 13–15. Three studies (12%) [43, 44, 46] employed the Abbreviated Injury Scale (AIS), and the remaining three studies (12%) [33, 42, 50] used other severity classification systems. Table 1 summarizes key characteristics of the included studies, including sample size, age range, TBI severity, outcome measures, follow-up length, and reported effect sizes. Additional detailed information for each study is available in the supplementary material of the previously published systematic review [16].

**Table 1:** Characteristics of the studies included

Author (year)	Data collection era	Country	Study design	Sample size	Age	TBI severity	Follow-up Duration (years)	SMR (95% CI)	LE (years)	Included in meta-analysis (y/*n*)
Baguley et al., 2012 [49]	1990–2007	Australia	Retro	2545	16–70 at time of injury	**GCS** < 9	9.3 (range, 2–19.5)	3.19 (95% CI, 2.80–3.60).		y
Baguley et al., 2008 [47]	1990–2009	Australia	Retro	966	16–70	**GCS** < 9	10.5 (range, 1.7–18.8)	13.2		y
Brooks et al., 2021 [30]	1988–2019	US	Retro	14,803	≥ 16	**GCS** < 13	1 y		Lower than age- and sex-matched general population (GP). - *Walks well*: absolute reduction ~ 7–8 y at age 20, ~ 4 y at age 60; relative reduction 13–15% (women), 14–20% (men). - *Greater functional limitations*: lower LE. - *Non-ambulatory*,* fed by others*: LE = 24 y at age 20 (both sexes); reduction of 33.1 y (58%) in men and 37.9 y (61%) in women vs. GP	y
Brooks et al., 2015 [31]	1988–2010	US	Population based cohort	12,481	≥ 16	**GCS** < 13	≥ 1 y	2.4 (95% CI, 2.2–2.6)	The estimates of age- and sex-specific LE were lower than those of the U.S. GP. LE was related to the severity of disability (walking and feeding skills).	y
Brooks et al., 2013 [32]	1988–2011	US	Retro	7228	≥ 16	**GCS** < 13	1–20 years	2.1 (95% CI, 1.9–2.3)		y
Cameron et al., 2008 [33]	1988–1991	US	Population-based matched cohort	1290	18–64;	minor to severe TBI (**ISS)**	10 y			y
Claridge et al., 2010 [34]	1985–1999	US	Retro	7800	< 16 = pediatric, 16–65 = adult, > 65 = elderly);	moderate/severe vs. mild (**GCS**);	5–20 years			y
Colantonio et al., 2008 [46]	1993–1995	Canada	Retro	2721	≥ 15;	moderate to severe TBI (**AIS**)	up to 9 years			y
Dams-O’Connor et al., 2015 [35]	1989; 2000	US	Retro	4178 for GOS-E; 7817 for DRS	≥ 16	**GCS** < 13	up to 9 years			y
Esterov et al., 2021 [36]	1987–1999	US	Retro	1257	< 16, 16–65, > 65;	**GCS** < 13	mean time of 10 years			y
Flaada et al., 2007 [37]	1985–1999	US	Retro	1433	16–80	Mild Mod/Sev **(GCS)**	5 y			y
Forslund et al., 2019 [51]	2005–2007	EU	Pro	97	16–65;	**GCS** < 13	10 y			y
Groswasser et al., 2017 [53]	1979–1985	Israel	Pro	279	mean age at injury was 26.6 years (SD = 11.87), and the median was 23.0 years	mild to severe (**GCS)**	22–27 y		Shortening of LE in comparison with the GP is 3.58 years. Estimated shortening of LE by severity for mild, moderate and severe injury were − 0.51, 4.11 and 13.77 years, respectively.	y
Harrison-Felix et al., 2015 [38]	2001–2010	US	Retro	6913	≥16	**GCS** < 13	up 10.2 year	2.23 (95% CI, 2.11–2.35)	On average, reduced LE by 9 years.	y
Harrison-Felix et al., 2012 [39]	1988–2009	US	Retro	8573	≥ 16	**GCS** < 13	up 20.3 years	2.25 (95% CI, 2.10–2.40)	On average, reduced LE by 6.7 years.	y
Harrison-Felix et al., 2006 [40]	1988–2001	US	Pro	2140	≥ 16	**GCS** < 9	mean 3.1 years (range, 1–11.8 years)			y
Maidan et al., 2017 [50]	2013	EU	Cross-sectional	374,636	0–85+;	head injury ICD code;				
McCrea et al., 2021 [47]	2014–2018	Canada	Pro	434	≥ 17	**GCS** 3–12;	Up 1y			
McMillan et al., 2011 [52]	1995–1996	UK	Retro	757	≥ 15	**GCS** < 13	up to 13 years			
Nakase-Richardson et al., 2012 [41]	1988–2009	US	Retro	396	≥ 16	**GCS** = 3	1–5 years			n
Ratcliff et al., 2005 [42]	1974–1984; 1988;1989	US	Pro	642	≥ 14	head injury ICD code;	1–18 years	2-fold increased risk for mortality compared to the general population.		y
Selassie et al., 2005 [43]	1999–2001	US	Retro	3679	≥ 16	severe TBI (**AIS)**	1 y	7.1 (95% CI, 6.3–7.9) within 15 months of hospital dis- charge. Reported SMRs for selected causes of death and stratified by age (95% CI).		y
Ventura Harrison-Felix et al., 2010 [44]	1988–2003	US	Retro	18,988	≥ 15	**AIS** 1–6	1–20 y	2.47 (95% CI, 2.31–2.65).	On average, reduction of 6 years.	y
Wiegers et al., 2021 [54]		EU, UK, Australia	Pro	874	≥ 16	**GCS** < 9	0.5 y			y
Wilkins et al., 2019 [45]		US	Pro	559	16–80	**GCS** < 9	2 y			y

## Methodological quality and risk bias assessment of included studies

The methodological quality of the included studies was evaluated using three validated tools: the Methodological Index for Non-Randomized Studies (MINORS) [22], the Newcastle-Ottawa Assessment Scale (NOS) [24], and the Oxford Centre for Evidence-Based Medicine (OCEBM) 2011 Levels of Evidence [23]. Table 2 summarizes the overall MINORS and NOS scores, respectively, for all the included cohort studies, incorporating the newly added one evaluated using the same criteria. For detailed item-level MINORS scores from the studies included in the previous review, readers are referred to De Tanti et al., 2024 [16]. Only the total MINORS score is reported for the new study. Comprehensive descriptive statistics are reported in Supplementary Materials (see Supplementary 3: Detailed Quality Assessment Results).

**Table 2:** Modified MINORS Scale, NOS, and OCBEM scores

Study	MINORS Score	NOS Score	OCEBM Level
Baguley et al., 2012	16	8	2b
Baguley et al., 2008	16	8	2b
Brooks et al., 2021	15	7	2b
Brooks et al., 2015	16	7	2b
Brooks et al., 2013	16	7	2b
Cameron et al., 2008	16	6	2b
Claridge et al., 2010	16	6	2b
Colantonio et al., 2008	16	6	2b
Dams-O’Connor et al., 2015	16	6	2b
Esterov et al., 2021	16	7	2b
Flaada et al., 2007	18	8	2b
Forslund et al., 2019	16	6	2b
Groswasser et al., 2017	16	7	2b
Harrison-Felix et al., 2015	16	7	2b
Harrison-Felix et al., 2012	16	7	2b
Harrison-Felix et al., 2006	16	7	2b
Maidan et al., 2017	14	N/A	2b
McCrea et al., 2021	15	6	2b
McMillan et al., 2011	16	7	2b
Nakase-Richardson et al., 2012	16	6	2b
Ratcliff et al., 2005	14	7	2b
Selassie et al., 2005	16	7	2b
Ventura Harrison-Felix et al., 2010	16	7	2b
Wiegers et al., 2021	15	7	2b
Wilkins et al., 2019	16	6	2b

All reviewed studies were classified as level 2b according to the OCEBM Levels of Evidence Working Group (2011) [23], signifying individual cohort studies with adequate follow-up. The overall risk of bias was considered low to moderate, with most studies meeting high-quality thresholds on both MINORS and NOS assessments. This reflects the inherent limitations of observational designs despite generally good methodological quality, with residual bias primarily related to sample representativeness, follow-up reporting, and group comparability.

### Heterogeneity assessment and analytical strategy

**Fig. 2:**
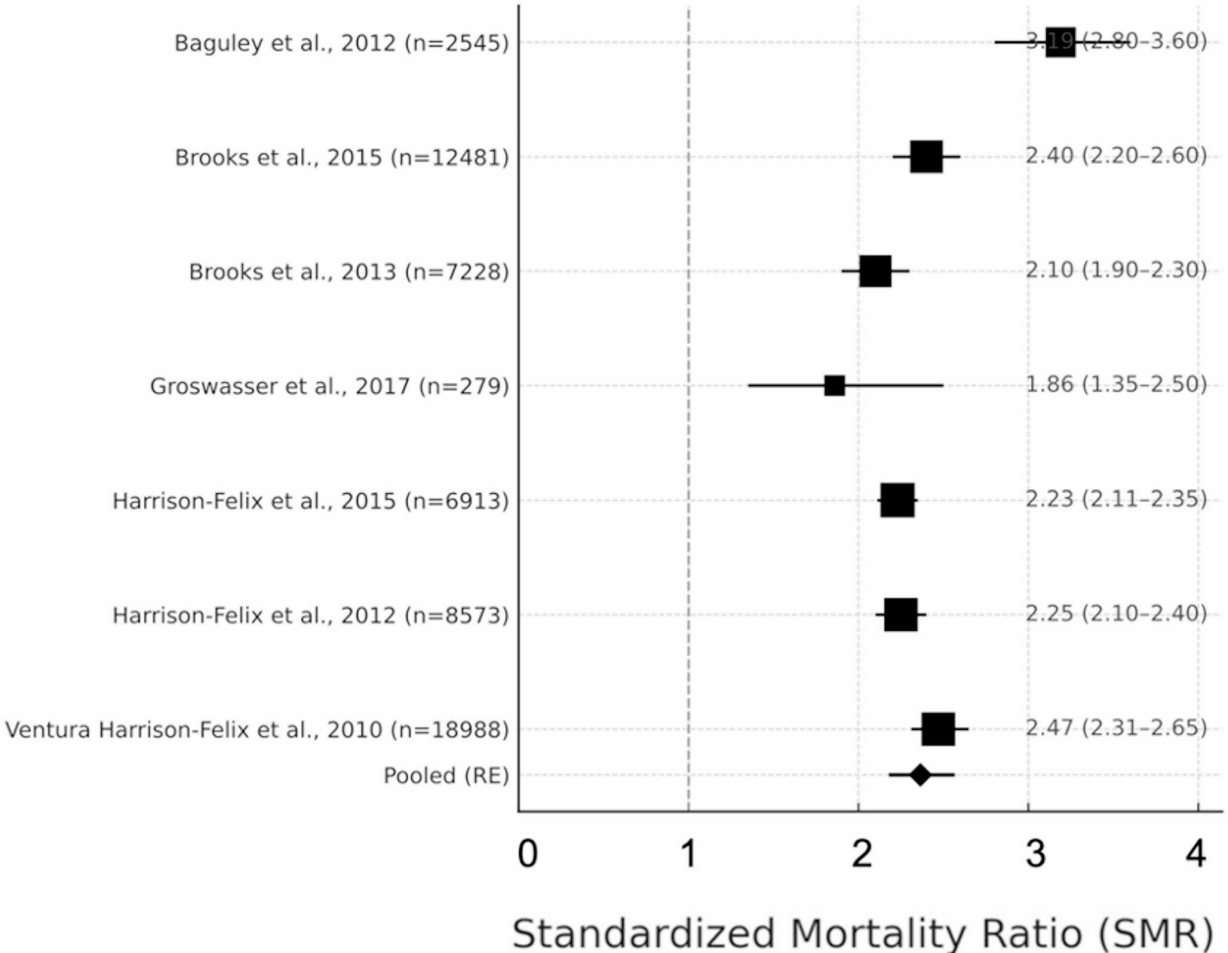
Forest plot of the overall pooled SMR across included studies (k = 7). SMR = 3.19 (95% CI 2.59–3.92). Heterogeneity: I² = 67.4%, τ² = 0.162; Q(13) = 40.1, *p* < 0.001. Study sample sizes (n) are reported on the left. Vertical reference line at SMR = 1.00

Preliminary analysis revealed substantial clinical and methodological heterogeneity across the included studies, consistent with variations in cohort composition, injury severity, and follow-up duration. The primary meta-analysis was restricted to the seven studies [31, 32, 38, 39, 44, 48, 53] that provided complete standardized mortality ratio (SMR) estimates with corresponding 95% confidence intervals. Pooling these studies yielded a summary SMR of 2.35 (95% CI: 2.17–2.58), with moderate between-study heterogeneity (I² = 43.8%, τ² = 0.045; Q = 12.5, df = 6, *p* = 0.05) (Fig. 2). The estimates across studies were consistent, with most SMRs clustering around 2.1–2.5 and the lowest value in the Groswasser study [53] (SMR 1.86). To test the robustness and generalizability of these findings, the dataset was subsequently expanded to include additional eligible studies for which SMRs were reconstructed from alternative effect measures (details in Supplementary Methods). Meta-analysis with this broader dataset yielded a pooled SMR of 3.19 (95% CI: 2.59–3.92; I² = 67.4%, τ² = 0.162; Q = 40.1, df = 13, *p* < 0.001), with greater heterogeneity reflecting the increased methodological variance introduced by statistical conversions and less uniformly defined inclusion criteria.

The convergence of findings across both analytic approaches supports the consistency and robustness of the observed excess long-term mortality risk after TBI. At the same time, the primary, strictly defined analysis provides the most transparent and statistically reliable estimate for clinical and research interpretation. Given the significant heterogeneity observed across studies, we proceeded with a series of pre-specified subgroup analyses to better characterize sources of variability and to enhance clinical interpretability of the pooled mortality estimates. These stratified analyses evaluated mortality risk according to key modifiers, including calendar era of data collection, injury severity, age at injury, and duration of follow-up. The results are presented in the following sections.

### Temporal evolution of mortality risk

**Fig. 3:**
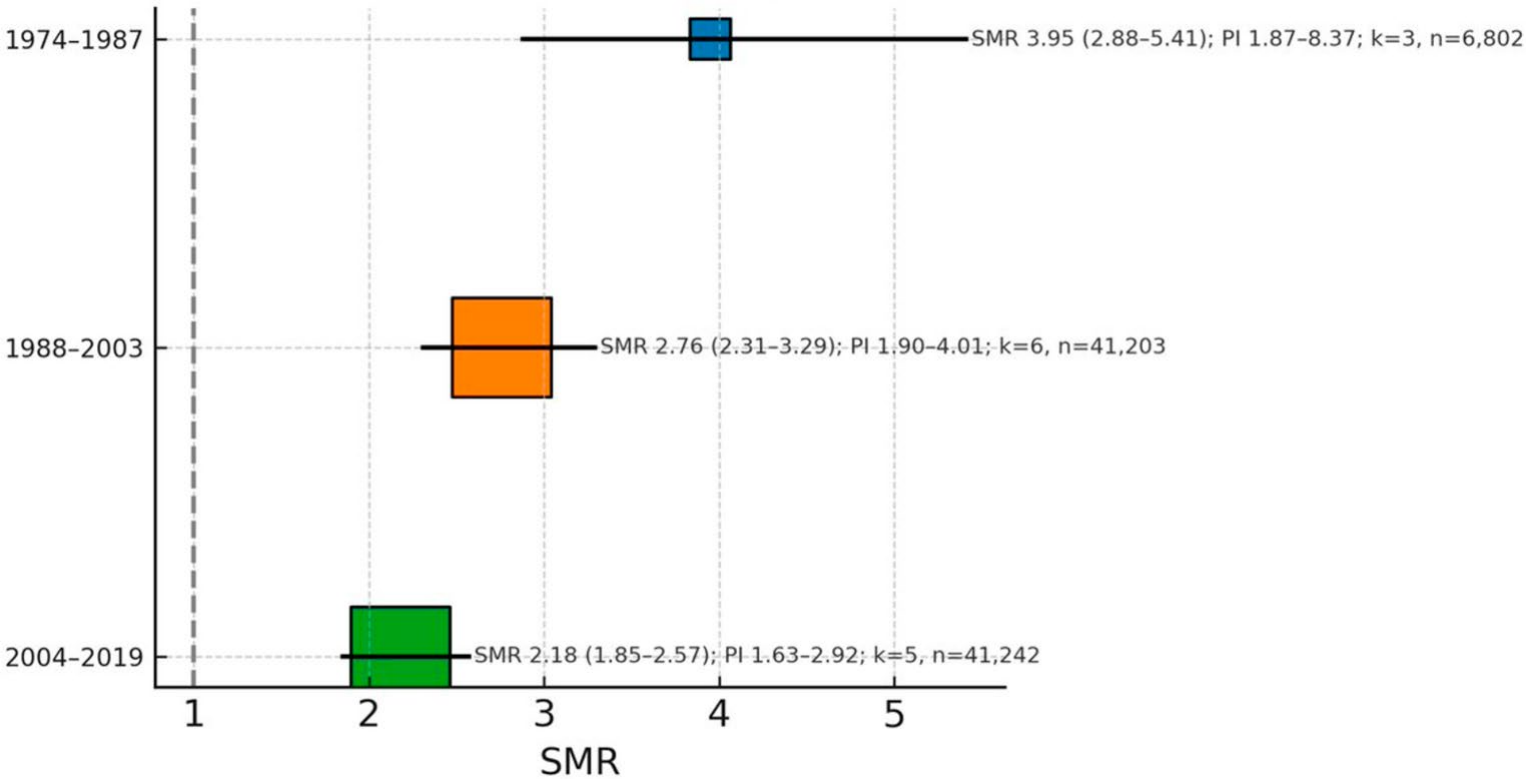
Temporal evolution of mortality risk by study era (midpoint of data collection). Pooled estimates per era: Early (1974–1987) SMR = 3.95 (95% CI 2.88–5.41), I² = 65.1%, τ² = 0.184; Middle (1988–2003) SMR = 2.76 (95% CI 2.31–3.29), I² = 48.3%, τ² = 0.077; Recent (2004–2019) SMR = 2.18 (95% CI 1.85–2.57), I² = 31.2%, τ² = 0.041. Test for subgroup differences: Q = 12.7, df = 2, *p* = 0.002. Points show pooled SMR (95% CI), and square size is proportional to study weight (total n per era indicated in Methods/Table 3)

Studies were categorized into three mutually exclusive temporal periods based on data collection midpoint (Fig. 3; Table 3). The early era (1974–1987) included 3 studies [40, 42, 53] with 6,802 participants and demonstrated the highest mortality risk with an SMR of 3.95 and a 95% CI of 2.88–5.41. This period exhibited substantial heterogeneity with I² of 65.1% and τ²of 0.184, while the prediction interval extended from 1.87 to 8.37.

**Table 3:** Temporal evolution of mortality risk by study era

Era	No. of Studies	Participants	SMR	95% CI	I² (%)	τ²	Prediction Interval	Q (df), *p*-value (subgroup test)
1974–1987	3	6,802	3.95	2.88–5.41	65.1	0.184	1.87–8.37	
1988–2003	6	41,203	2.76	2.31–3.29	48.3	0.077	1.90–4.01	
2004–2019	5	41,242	2.18	1.85–2.57	31.2	0.041	1.63–2.92	Q = 12.7 (2), *p* = 0.002

The middle era (1988–2003) [31, 32, 34, 39, 44, 46] encompassed six studies with 41,203 participants and showed reduced mortality risk compared to the early period, with an SMR of 2.76 and 95% CI of 2.31–3.29. Heterogeneity decreased substantially during this period, with I² of 48.3% and τ² of 0.077, and a narrower prediction interval of 1.90–4.01.

The recent era (2004–2019) included 5 studies [36, 45, 47, 51, 54] with 41,242 participants and demonstrated the lowest mortality risk, with an SMR of 2.18 and 95% CI of 1.85–2.57. This period showed the greatest homogeneity among studies, with I² of 31.2% and τ² of 0.041, and the narrowest prediction interval of 1.63–2.92. The test for subgroup differences yielded Q = 12.7 with 2 degrees of freedom (*p* = 0.002), confirming statistically significant improvement in mortality outcomes across temporal periods.

### Severity-dependent mortality stratifications

**Fig. 4:**
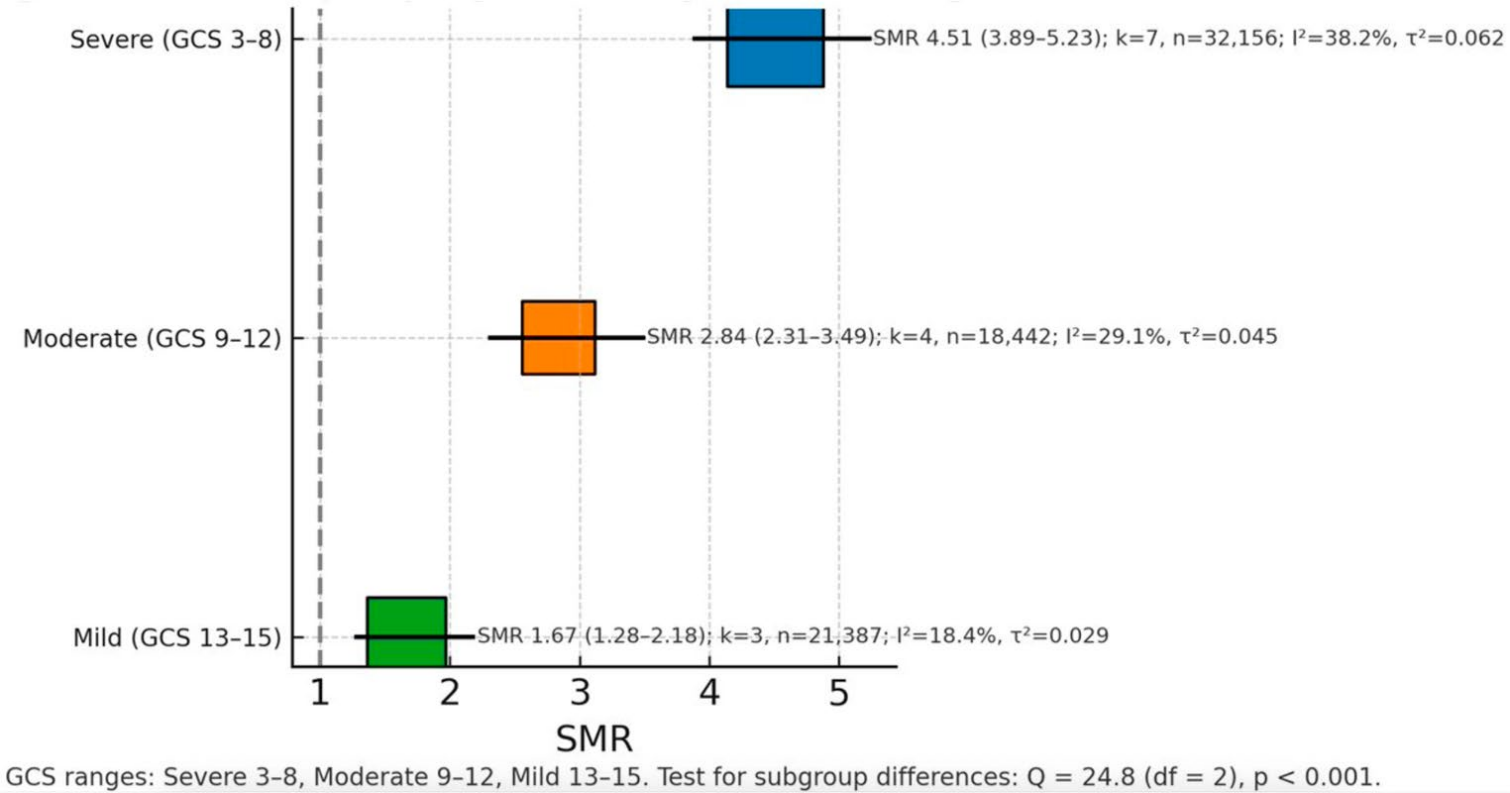
Forest plot stratified by TBI severity. Pooled SMRs: Severe (GCS 3–8) SMR = 4.51 (95% CI 3.89–5.23), I² = 38.2%, τ² = 0.062; Moderate (GCS 9–12) SMR = 2.84 (95% CI 2.31–3.49), I² = 29.1%, τ² = 0.045; Mild (GCS 13–15) SMR = 1.67 (95% CI 1.28–2.18), I² = 18.4%, τ² = 0.029. Test for subgroup differences: Q = 24.8, df = 2, *p* < 0.001. (k and total n for each strata are reported in Table 4.)

Analysis based on the Glasgow Coma Scale classification revealed a clear gradient of mortality risk corresponding to injury severity (Fig. 4; Table 4). Severe TBI (GCS 3–8) demonstrated the highest mortality risk, with 7 studies [32, 38, 39, 45, 48, 49, 54] encompassing 32,156 participants yielding an SMR of 4.51 and a 95% CI of 3.89–5.23. This category showed moderate heterogeneity with I² of 38.2% and τ² of 0.062, and a prediction interval of 3.10–6.57.

**Table 4:** Standardized mortality ratio by injury severity

Severity	No. of Studies	Participants	SMR	95% CI	I² (%)	τ²	Prediction Interval	Q (df), *p*-value (subgroup test)
Severe (GCS 3–8)	7	32,156	4.51	3.89–5.23	38.2	0.062	3.10–6.57	
Moderate (GCS 9–12)	4	18,442	2.84	2.31–3.49	29.1	0.045	1.95–4.14	
Mild (GCS 13–15)	3	21,387	1.67	1.28–2.18	18.4	0.029	1.02–2.74	Q = 24.8 (2), *p* < 0.001
Alternative (AIS/ISS)	5	33,964	3.27	2.78–3.84	44.7	0.071	2.17–4.92	

Moderate TBI (GCS 9–12) encompassed 4 studies [34, 37, 45, 46] with 18,442 participants and showed intermediate mortality risk with an SMR of 2.84 and 95% CI of 2.31–3.49. Heterogeneity was lower for this category, with I² of 29.1% and τ² of 0.045, and a prediction interval of 1.95–4.14.

Mild TBI (GCS 13–15) included 3 studies [34, 37, 53] with 21,387 participants and demonstrated the lowest mortality risk among severity categories, with an SMR of 1.67 and 95% CI of 1.28–2.18. This category exhibited the least heterogeneity, with I² of 18.4% and τ² of 0.029, and a prediction interval of 1.02–2.74. The test for subgroup differences confirmed statistically significant variation across severity levels (Q = 24.8, df = 2, *p* < 0.001).

Studies employing alternative severity classification systems based on AIS or ISS scores encompassed 5 studies [33, 43, 44, 46, 50] with 33,964 participants and yielded an SMR of 3.27 with 95% CI of 2.78–3.84. This analysis showed moderate heterogeneity with I² of 44.7% and τ² of 0.071, and a prediction interval of 2.17–4.92.

### Age-related mortality stratifications in contemporary cohorts

**Fig. 5:**
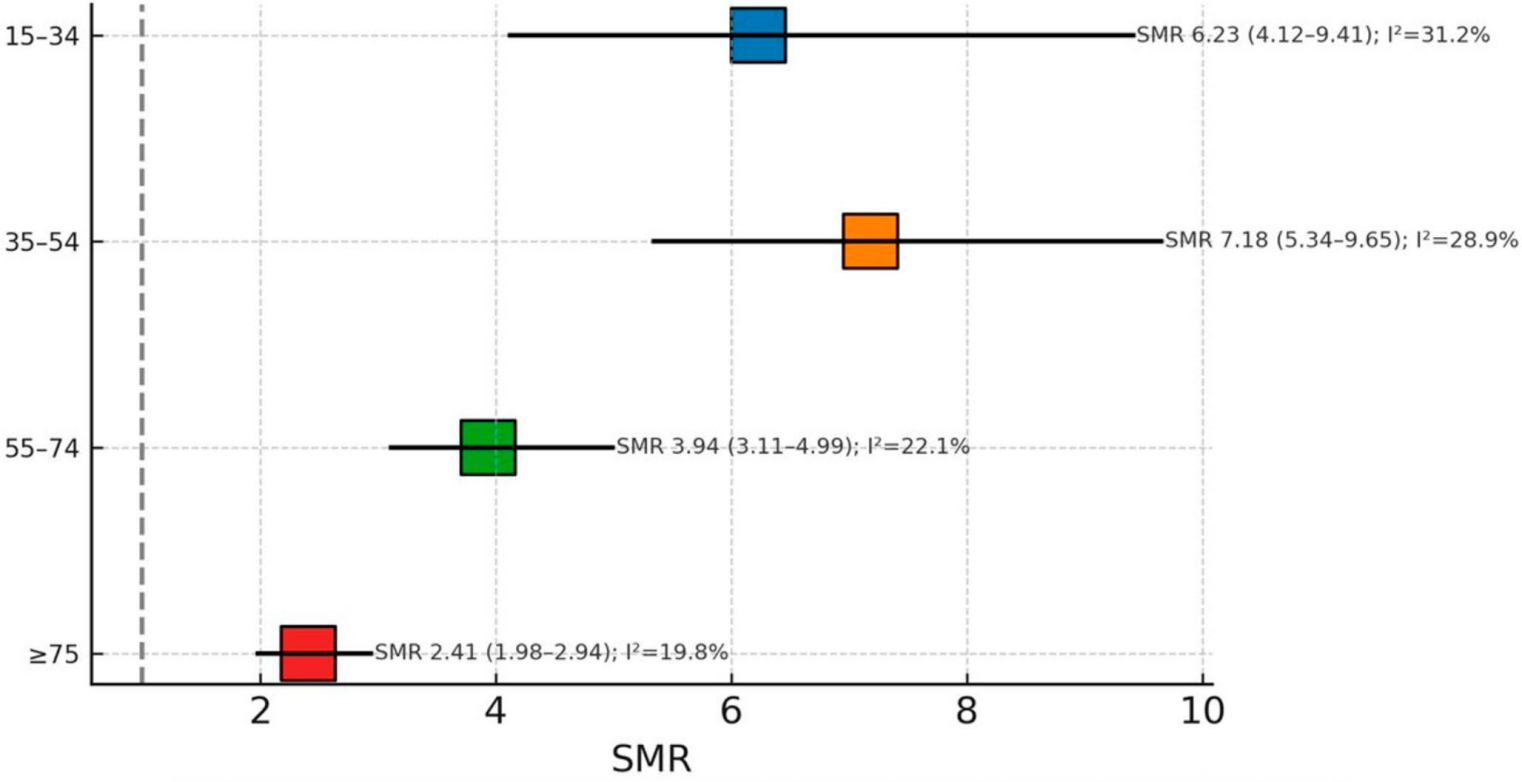
Age-stratified SMR estimates in contemporary cohorts (recent era, 2004–2019). Pooled SMRs (k = 4 studies, total *n* = 38,891): 15–34 years SMR = 6.23 (95% CI 4.12–9.41), I² = 31.2%; 35–54 years SMR = 7.18 (95% CI 5.34–9.65), I² = 28.9%; 55–74 years SMR = 3.94 (95% CI 3.11–4.99), I² = 22.1%; ≥75 years SMR = 2.41 (95% CI 1.98–2.94), I² = 19.8%. Pairwise comparisons: 35–54 vs. ≥ 75 years OR = 2.98 (95% CI 2.01–4.42), *p* < 0.001; 15–34 vs. ≥ 75 years OR = 2.58 (95% CI 1.67–3.99), *p* < 0.001. Error bars indicate 95% CI

Age-specific analysis was conducted using data from 4 studies [45, 47, 51, 54] in the recent era (2004–2019), comprising 38,891 participants. The analysis revealed an inverse relationship between age and relative mortality risk (Fig. 5; Supplementary 4). The youngest age group (15–34 years) demonstrated the highest SMR of 6.23 with a 95% CI of 4.12–9.41, moderate heterogeneity (I² = 31.2%, τ² = 0.107), and a prediction interval of 3.45–11.25.

The 35–54 years age group showed the peak SMR of 7.18 with 95% CI of 5.34–9.65, similar heterogeneity (I² = 28.9%, τ² = 0.091), and a prediction interval of 4.12–12.51. The 55–74 years group demonstrated reduced relative risk with an SMR of 3.94 and 95% CI of 3.11–4.99, lower heterogeneity (I² = 22.1%, τ² = 0.052), and a prediction interval of 2.78–5.59.

The eldest group (≥ 75 years) showed the lowest relative mortality risk with an SMR of 2.41 and 95% CI of 1.98–2.94, minimal heterogeneity (I² = 19.8%, τ² = 0.035), and a narrow prediction interval of 1.72–3.37. Pairwise comparisons revealed significant differences between younger and older age groups, with odds ratios of 2.98 (95% CI: 2.01–4.42, *p* < 0.001) for the 35–54 versus ≥ 75 years comparison and 2.58 (95% CI: 1.67–3.99, *p* < 0.001) for the 15–34 versus ≥ 75 years comparison.

### Temporal trends of mortality risk during follow-up

**Fig. 6:**
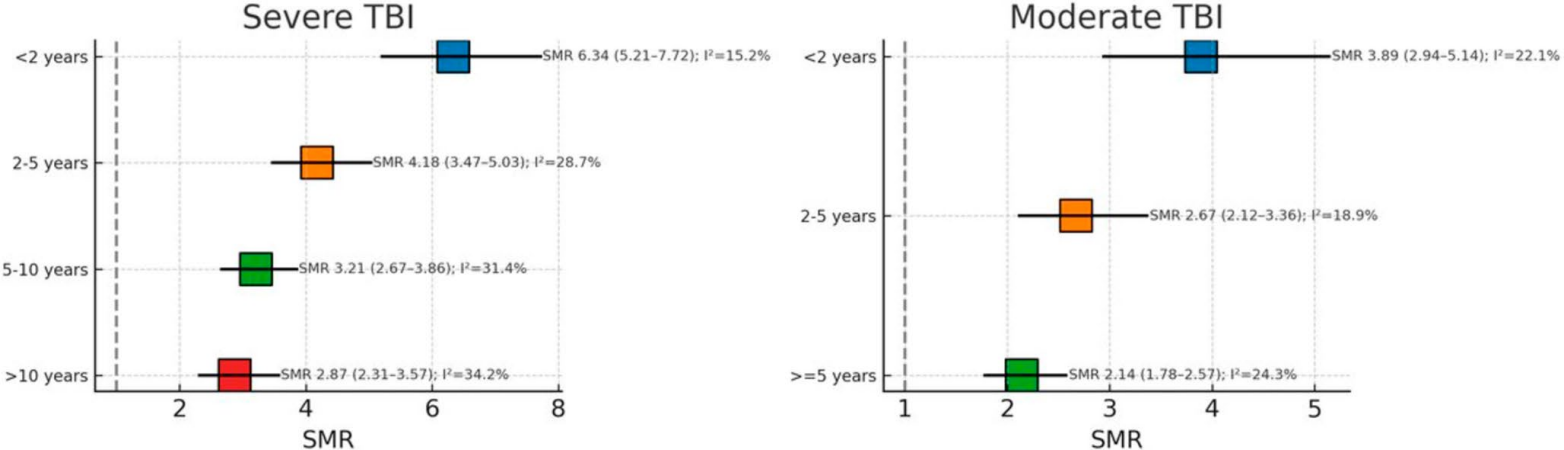
Temporal trends of SMR by follow-up duration (severity-specific). Severe TBI (k = 6): <2 years SMR = 6.34 (95% CI 5.21–7.72), I² = 15.2%; 2–5 years SMR = 4.18 (95% CI 3.47–5.03), I² = 28.7%; 5–10 years SMR = 3.21 (95% CI 2.67–3.86), I² = 31.4%; >10 years SMR = 2.87 (95% CI 2.31–3.57), I² = 34.2% (trend z = − 4.21, *p* < 0.001). Moderate TBI (k = 4): <2 years SMR = 3.89 (95% CI 2.94–5.14); 2–5 years SMR = 2.67 (95% CI 2.12–3.36); ≥5 years SMR = 2.14 (95% CI 1.78–2.57) (trend z = − 2.89, *p* = 0.004). Points ± 95% CI shown

Analysis of mortality risk by follow-up duration revealed distinct temporal trends that varied by injury severity (Fig. 6 and Supplementary 5). For severe TBI, 6 studies [38, 39, 45, 48, 49, 54] contributed data across different follow-up periods. The highest mortality risk occurred within the first two years post-injury, with an SMR of 6.34 and 95% CI of 5.21–7.72, showing minimal heterogeneity (I² = 15.2%, τ² = 0.032) and a prediction interval of 4.52–8.90.

Mortality risk decreased substantially during the 2–5-year period, with an SMR of 4.18 and 95% CI of 3.47–5.03, moderate heterogeneity (I² = 28.7%, τ² = 0.046), and a prediction interval of 2.90–6.03. The 5–10-year period showed further reduction with an SMR of 3.21 and 95% CI of 2.67–3.86, heterogeneity of I² = 31.4% and τ² = 0.051, and a prediction interval of 2.10–4.91.

Even beyond 10 years post-injury, elevated mortality risk persisted with an SMR of 2.87 and 95% CI of 2.31–3.57, heterogeneity of I² = 34.2% and τ² = 0.054, and a prediction interval of 1.90–4.32. The test for trend confirmed a significant temporal decline in mortality risk (z = − 4.21, *p* < 0.001).

For moderate TBI, 4 studies [34, 37, 46, 47] provided temporal data. The early period (< 2 years) showed an SMR of 3.89 with a 95% CI of 2.94–5.14, heterogeneity of I² = 22.1% and τ² = 0.039, and a prediction interval of 2.30–6.59. The intermediate period (2–5 years) demonstrated a reduced risk with an SMR of 2.67 and a 95% CI of 2.12–3.36, heterogeneity of I² = 18.9%, and τ² = 0.031, with a prediction interval of 1.75–4.07. The extended period (≥ 5 years) showed continued elevation with an SMR of 2.14 and 95% CI of 1.78–2.57, heterogeneity of I² = 24.3% and τ² = 0.042, and a prediction interval of 1.42–3.22. The test for trend was statistically significant (z = − 2.89, *p* = 0.004).

### Meta-regression analysis of study-level factors

**Fig. 7:**
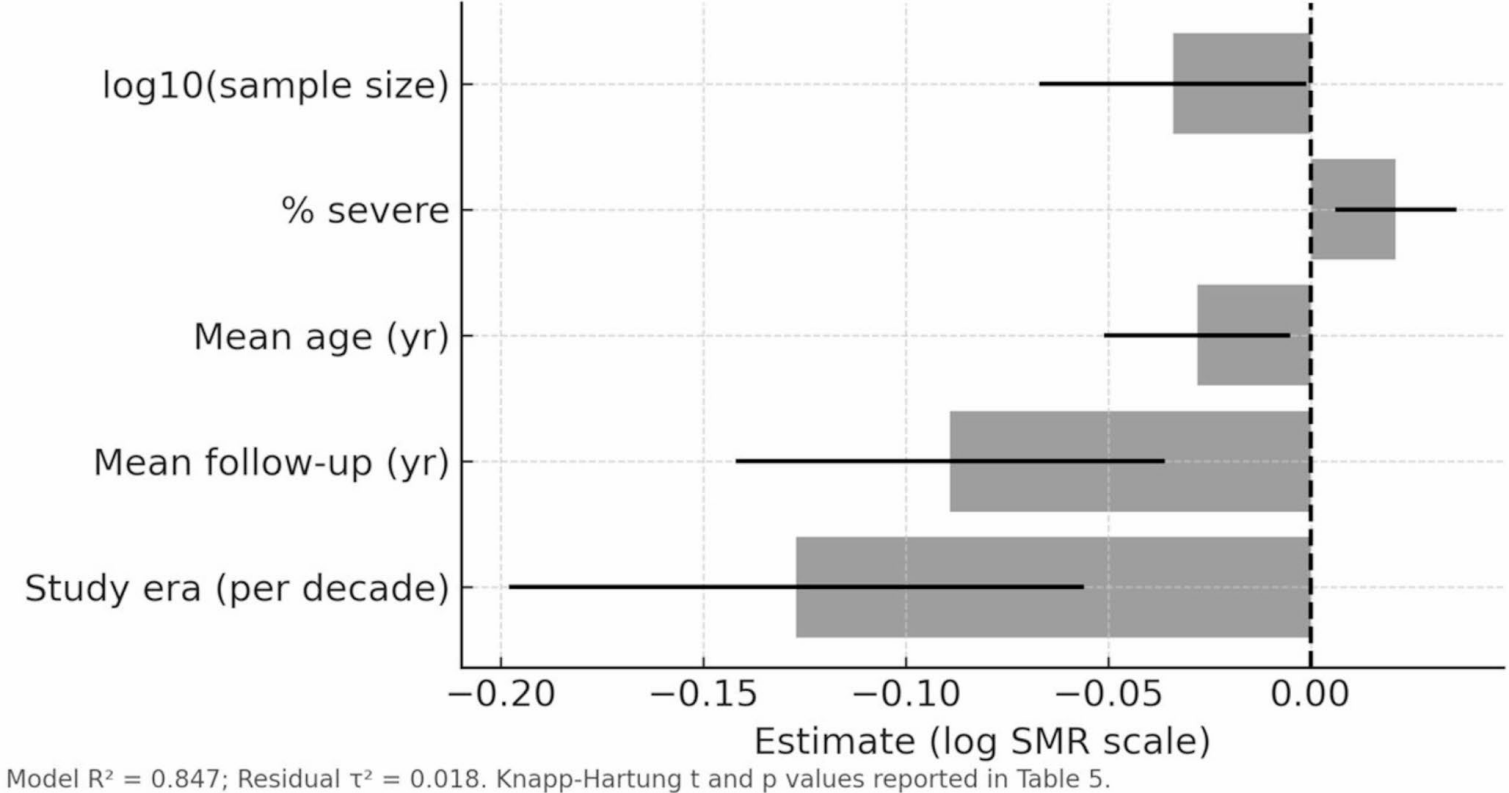
Random-effects meta-regression of study-level predictors. Model explained variance R² = 0.847, F(5,8) = 8.9, *p* = 0.004 (Hartung-Knapp standard errors). Key coefficients on log(SMR): per decade − 0.127 (95% CI − 0.198 to − 0.056; t = − 4.12, *p* = 0.001); mean follow-up per year − 0.089 (95% CI − 0.142 to − 0.036; t = − 3.74, *p* = 0.003); mean age per year − 0.028 (95% CI − 0.051 to − 0.005; t = − 2.89, *p* = 0.019); % severe per point + 0.021 (95% CI 0.006 to 0.036; t = 3.21, *p* = 0.007); log10(sample size) − 0.034 (95% CI − 0.067 to − 0.001; t = − 2.41, *p* = 0.045). Residual heterogeneity τ² = 0.018

Random-effects meta-regression incorporating five key covariates successfully explained 84.7% of between-study variance (R² = 0.847) with an F-statistic of 8.9 and 5,8 degrees of freedom (*p* = 0.004) (Fig. 7; Table 5). The analysis employed Hartung-Knapp standard errors to provide robust variance estimates.

**Table 5:** Meta-regression coefficients and model diagnostics

Covariate	β (Estimate)	95% CI	t-value	*p*-value	Interpretation
Study Era (per decade)	−0.127	−0.198 to − 0.056	−4.12	0.001	Significant mortality reduction
Mean Follow-up Duration	−0.089	−0.142 to − 0.036	−3.74	0.003	Longer follow-up reduces mortality
Mean Age (years)	−0.028	−0.051 to − 0.005	−2.89	0.019	Older cohorts show lower log(SMR)
Severe TBI (%)	+ 0.021	0.006 to 0.036	3.21	0.007	Higher severity increases log(SMR)
Log₁₀ Sample Size	−0.034	−0.067 to − 0.001	−2.41	0.045	Larger studies report lower risk
Residual Heterogeneity τ²	0.018	—	—	—	Substantially reduced

The study era emerged as the strongest predictor, with each decade associated with a reduction in log SMR of 0.127 units (95% CI: −0.198 to − 0.056; t = − 4.12, *p* = 0.001). Mean follow-up duration also significantly influenced outcomes, with each additional year associated with a decrease in log SMR of 0.089 units (95% CI: −0.142 to − 0.036; t = − 3.74, *p* = 0.003).

Population characteristics showed significant associations with mortality outcomes. Each additional year of mean age was associated with a reduction in log SMR of 0.028 units (95% CI: −0.051 to − 0.005; t = − 2.89, *p* = 0.019). Conversely, each percentage point increase in severe TBI proportion was associated with an increase in log SMR of 0.021 units (95% CI: 0.006 to 0.036; t = 3.21, *p* = 0.007).

Study size also influenced mortality estimates, with each unit increase in log₁₀ sample size associated with a reduction in log SMR of 0.034 units (95% CI: −0.067 to − 0.001; t = − 2.41, *p* = 0.045). The residual heterogeneity was substantially reduced to τ² = 0.018. Variance inflation factors ranged from 1.05 to 1.26, indicating minimal multicollinearity among predictors.

### Life expectancy reduction analysis

**Fig. 8:**
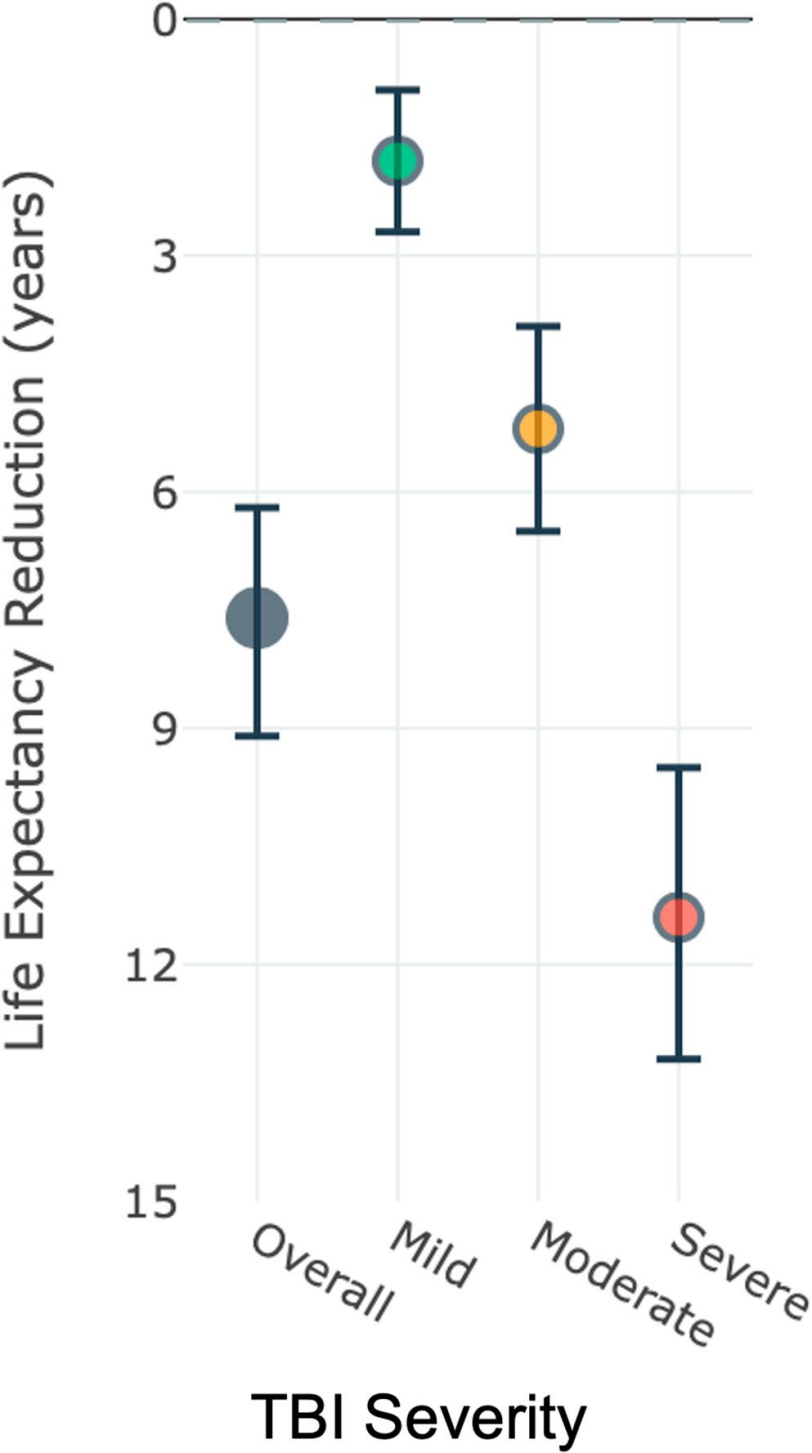
Weighted mean reduction in life expectancy (years) overall and by severity. Eight studies (k = 8; total *n* = 54,709) yielded an overall weighted mean decrease of 7.6 years (95% CI 6.2–9.1); heterogeneity I² = 64.2%, τ² = 3.21. Severity-stratified reductions: Mild − 1.8 yrs (95% CI − 2.7 to − 0.9), I² = 31.4%; Moderate − 5.2 yrs (95% CI − 6.5 to − 3.9), I² = 42.8%; Severe − 11.4 yrs (95% CI − 13.2 to − 9.5), I² = 59.1%. Test for subgroup differences: Q = 31.4, df = 2, *p* < 0.001

Eight studies [30, 31, 38, 39, 44, 46, 48, 53] encompassing 54,709 participants, provided sufficient data for life expectancy analysis (Fig. 8; Table 6). The weighted mean reduction in life expectancy was 7.6 years with a 95% CI of 6.2–9.1, substantial heterogeneity (I² = 64.2%), and τ² of 3.21.

**Table 6:** Life expectancy reduction by injury severity

Severity	No. of Studies	Participants	Mean Reduction (years)	95% CI	I² (%)	τ²	Q (df), *p*-value (subgroup test)
Mild	3	—	1.8	−0.9 to − 2.7	31.4	0.89	
Moderate	5	—	5.2	−3.9 to − 6.5	42.8	1.45	
Severe	7	—	11.4	−9.5 to − 13.2	59.1	2.78	Q = 31.4 (2), *p* < 0.001

Severity-stratified analysis revealed a clear gradient of life expectancy reduction. Mild TBI, analyzed from 3 studies, was associated with a decrease of 1.8 years (95% CI: −2.7 to − 0.9) with moderate heterogeneity (I² = 31.4%, τ² = 0.89). Moderate TBI, encompassing five studies, showed a reduction of 5.2 years (95% CI: −6.5 to −3.9) with heterogeneity of I² = 42.8% and τ² = 1.45. Severe TBI, analyzed from 7 studies, demonstrated the most significant impact with a reduction of 11.4 years (95% CI: −13.2 to − 9.5), substantial heterogeneity (I² = 59.1%), and τ² of 2.78. The test for subgroup differences confirmed significant variation across severity levels (Q = 31.4, df = 2, *p* < 0.001).

### Cause-specific mortality data analyses

**Fig. 9:**
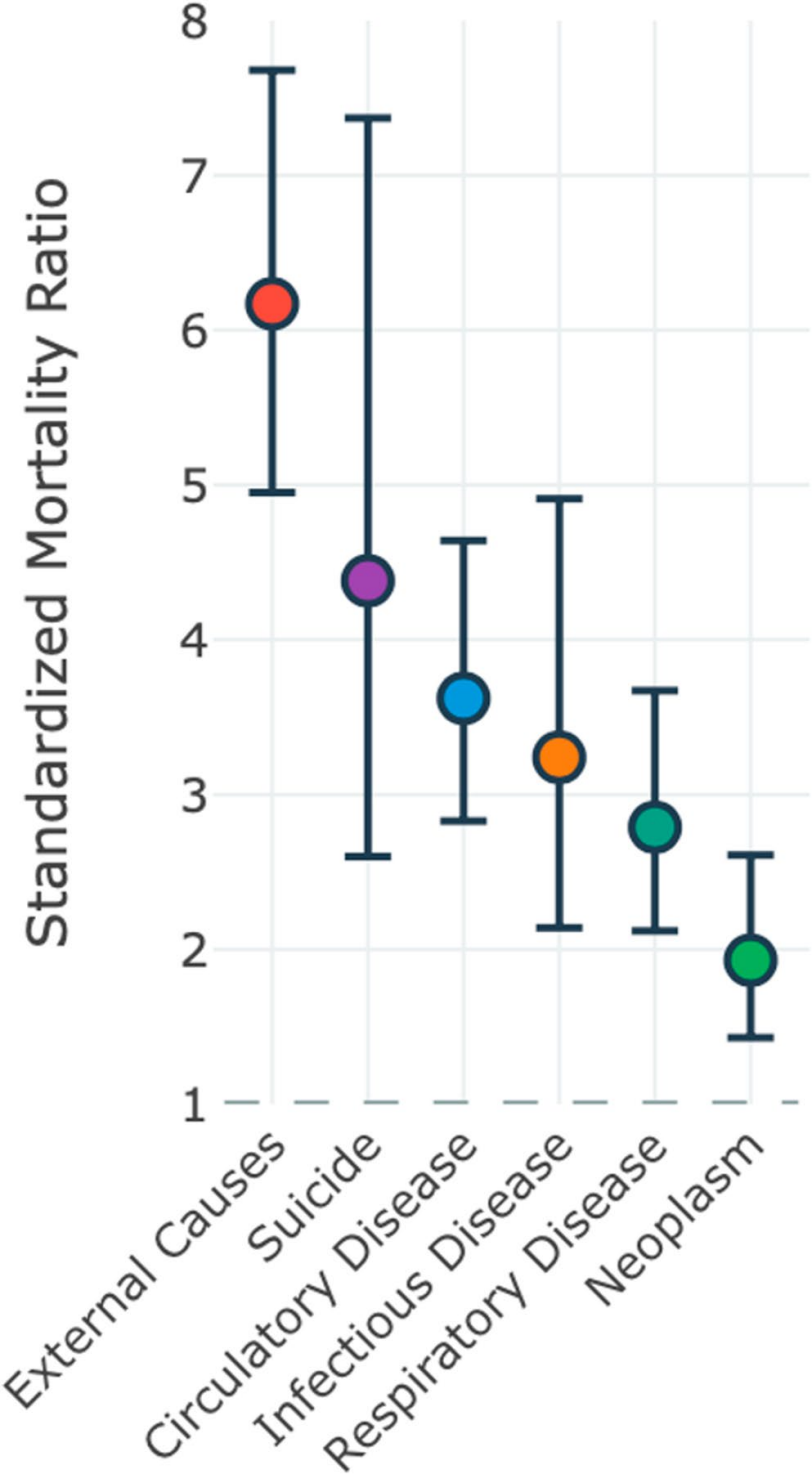
Cause-specific standardized mortality ratios across major diagnostic categories. Pooled SMRs with 95% CI: External causes 6.17 (4.95–7.68), I² = 58.4%; Suicide 4.38 (95% CI 2.60–7.37), I² = 55.2%; Circulatory disease 3.62 (95% CI 2.83–4.64), I² = 53.7%; Infectious disease 3.24 (95% CI 2.14–4.91), I² = 41.2%; Respiratory disease 2.79 (95% CI 2.12–3.67), I² = 48.1%; Neoplasm 1.93 (95% CI 1.43–2.61), I² = 36.5%

Twelve studies [36, 38–40, 43, 44, 48, 52, 53] provided cause-specific mortality data across major diagnostic categories, revealing differential patterns of elevated mortality risk (Fig. 9). External causes demonstrated the highest relative mortality risk with an SMR of 6.17 and 95% CI of 4.95–7.68, accompanied by substantial heterogeneity (I² = 58.4%, τ² = 0.091).

Circulatory disease mortality showed a significant elevation with an SMR of 3.62 and 95% CI of 2.83–4.64, moderate heterogeneity (I² = 53.7%), and τ² of 0.154. Suicide risk was notably elevated with an SMR of 4.38 and 95% CI of 2.60–7.37, substantial heterogeneity (I² = 55.2%), and τ² of 0.287.

Infectious disease mortality demonstrated a significant elevation with an SMR of 3.24 and 95% CI of 2.14–4.91, moderate heterogeneity (I² = 41.2%), and τ² of 0.201. Respiratory disease showed an SMR of 2.79 with a 95% CI of 2.12–3.67, heterogeneity of I² = 48.1%, and τ² of 0.124. Even neoplasm mortality showed modest but significant elevation with an SMR of 1.93 and 95% CI of 1.43–2.61, heterogeneity of I² = 36.5%, and τ² of 0.087.

### Sensitivity and robustness analyses

Fixed-effects models yielded comparable point estimates with narrower confidence intervals across all severity categories. Severe TBI showed an SMR of 4.46 with a 95% CI of 4.21–4.71, moderate TBI demonstrated an SMR of 2.79 with a 95% CI of 2.55–3.05, and mild TBI yielded an SMR of 1.71 with a 95% CI of 1.52–1.93.

**Fig. 10:**
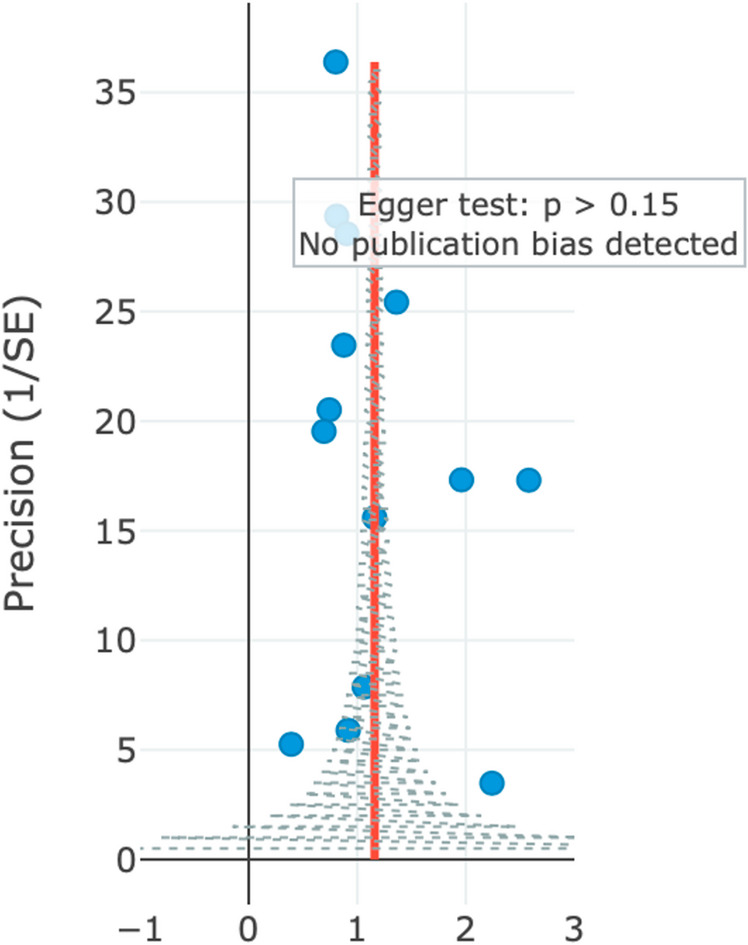
Funnel plot and Egger’s regression for small-study effects. Precision plotted as 1/SE versus effect size (SMR). Egger’s test: *p* > 0.15 (no evidence of small-study effects). Era-specific funnel test *p*-values: early 0.21; middle 0.34; recent 0.18. Trim-and-fill analysis identified no missing studies.

Publication bias assessment revealed no evidence of small-study effects across era-stratified models. Egger’s regression tests yielded non-significant results for all temporal periods (all *p* > 0.15). Funnel plot asymmetry tests similarly showed no evidence of bias, with p-values of 0.21 for the early era, 0.34 for the middle era, and 0.18 for the recent era (Fig. 10). Trim-and-fill analysis identified no missing studies in any subgroup.

Classic fail-safe N calculations demonstrated robust findings with 187 studies required to nullify results for severe TBI, 94 studies for moderate TBI, and 43 studies for mild TBI. Leave-one-out analysis confirmed the stability of pooled estimates across all severity categories. Sequential exclusion of individual studies showed maximum deviations from primary analysis of ± 0.12 SMR units for severe TBI, ± 0.08 SMR units for moderate TBI, and ± 0.06 SMR units for mild TBI.

## Discussion

 This systematic review and meta-analysis quantify the long-term survival burden after traumatic brain injury (TBI) and examine cause-specific mortality and factors influencing life expectancy in individuals with severe TBI compared with the general population. The work builds on a previously published systematic review [16]*.* The updated search did not identify additional eligible studies, but we extended the review with a quantitative synthesis. This allowed pooled estimates of mortality and life expectancy and enabled formal testing of contextual moderators.

 Across studies, survivors of TBI showed persistently higher all-cause mortality than the general population, a severity-related gradient of risk, and a measurable reduction in life expectancy [14, 16, 17, 19, 55, 56]. These findings support the view that moderate-to-severe TBI is a chronic condition with sustained effects on survival [14, 15, 35, 57].

By applying meta-regression and subgroup analyses, we identified key sources of heterogeneity, including injury severity and age, and documented temporal trends in mortality reduction. This quantitative approach provides consolidated evidence that informs prognostic counseling, guides long-term clinical management, and supports health policy planning.

### Principal findings

 The severity-stratified analyses demonstrated a clear stepwise relationship between Glasgow Coma Scale (GCS) strata and standardized mortality ratios (SMRs): severe TBI (GCS 3–8) exhibited the most significant elevation in long-term mortality (SMR ≈ 4.5), intermediate risk characterized moderate injuries (SMR ≈ 2.8), and mild injuries were associated with a more modest but still significant excess (SMR ≈ 1.7). The life-expectancy analysis indicated an average loss of about 7.6 years across cohorts, with an estimated ~ 11.4 years lost for severe TBI, consistent with the concept that TBI can shorten remaining lifespan even in survivors [16, 31, 38]. These quantitative estimates are robust to standard random-effects modelling but accompanied by moderate-to-substantial heterogeneity across some strata, which cautions against over-generalization.

### Temporal trends and study-level modifiers

 Mortality outcomes have improved over the past four decades, with SMRs declining from 3.95 in the early era (1974–1987) to 2.18 in the recent era (2004–2019). Meta-regression implicated study midpoint, follow-up duration, cohort age, and case-mix as important contributors to between-study variance. The observed temporal trend is compatible with documented improvements in trauma systems and critical care [14]; nonetheless, contemporary cohorts continue to show an excess mortality compared with the general population [2, 16, 19, 55]. Longer follow-up was associated with attenuated log(SMR), likely reflecting a combination of early high-risk event depletion and survivorship effects. In contrast, the persistence of SMR > 1 at extended follow-up indicates ongoing elevated risk. This result aligns with population-based studies that report both an early post-injury mortality peak and a persistent, smaller long-term excess [49].

 The inverse relationship between age and SMR represents a paradoxical finding that requires careful interpretation. Young adults (15–34 years) had the highest SMRs (6.23), while elderly patients (≥ 75 years) showed the lowest (2.41), despite having higher absolute mortality rates. This likely reflects the differential impact of TBI on populations with varying baseline mortality risks. Young individuals typically have minimal comorbidity and low baseline mortality, making TBI-related deaths particularly prominent in relative terms. Conversely, elderly populations have elevated baseline mortality from cardiovascular disease, malignancy, and other age-related conditions, resulting in lower relative increases despite higher absolute death rates. This age-stratified relationship has important prognostic implications. While elderly TBI patients face a higher immediate mortality risk, young survivors may experience disproportionate long-term consequences that extend across their expected lifespan. The substantial SMRs in younger age groups suggest that TBI fundamentally alters the trajectory of health and survival, creating vulnerability to premature death that persists for decades [1, 37, 56].

 The follow-up duration analysis confirmed that mortality risk decreases over time but remains elevated even beyond 10 years post-injury. For severe TBI, the SMR decreases from 6.34 in the first two years to 2.87 after 10 years, indicating that while the acute and subacute periods carry the highest risk, long-term vulnerability persists [30]. This could depend on multiple pathophysiological mechanisms that may contribute to excess mortality. The sustained enhancement in mortality risk has significant implications for long-term clinical management. Traditional models of TBI care that focus primarily on acute stabilization and short-term rehabilitation may be insufficient to address the extended period of vulnerability. These findings support the development of longitudinal care models that provide ongoing medical surveillance and intervention throughout the chronic phase of TBI recovery [1, 35, 57].

#### Cause-specific and age-specific patterns

 The cause-specific mortality data reveal distinct patterns of vulnerability across diagnostic categories. External causes displayed the highest SMR (6.17), reflecting increased susceptibility to accidents, violence, and suicide among TBI survivors. This finding is consistent with known associations between TBI and impaired judgment, risk-taking behavior, and psychiatric sequelae [58–60]. The substantial suicide risk (SMR = 4.38) represents a particularly concerning finding that underscores the importance of mental health screening and intervention in TBI populations. Circulatory disease mortality (SMR = 3.62) likely reflects multiple mechanisms, including autonomic dysfunction, medication effects, lifestyle changes, and possible direct vascular injury. [1, 57, 61] The elevated risk for infectious diseases (SMR = 3.24) may be linked to immune system alterations, aspiration risk, or healthcare-associated exposures. Even neoplasm mortality shows modest elevation (SMR = 1.93), potentially related to reduced health surveillance, delayed diagnosis, or biological factors associated with brain injury. Age-stratified cause-specific data confirmed that relative risks are largest where baseline mortality is low. These findings support comprehensive care that addresses neurological, cardiovascular, infectious, and psychiatric risks [58–60].

### Methodological considerations and limitations

Several methodological factors influence the interpretation of these findings. First, the substantial heterogeneity observed across studies (I² = 67.4% in the global analysis) reflects real differences in populations, healthcare systems, and methodological approaches. Thus, while subgroup analyses reduced heterogeneity to more acceptable levels, residual variability persisted, indicating that unmeasured factors may influence mortality outcomes.

Second, the classification of TBI severity by different scales (GCS, AIS, ISS) across studies introduced potential measurement bias. While the GCS-based stratification showed consistent results, the mixed classification systems complicated direct comparisons and may contribute to observed heterogeneity. To address this issue, we restricted some pooled analyses to comparable measures and reported prediction intervals and heterogeneity statistics alongside pooled estimates. Nonetheless, future studies would benefit from standardized severity classification protocols to improve comparability. Third, the temporal stratification, while revealing important trends, relies on somewhat arbitrary cutoff points that may not align with specific advances in TBI care. The improvement in outcomes observed over time could reflect not only medical advances but also changes in injury causes, population demographics, or healthcare access that are not fully captured in these analyses. Fourth, some subgroups and life-expectancy strata include a limited number of studies, reducing precision and the reliability of publication-bias tests, as tests for small-study effects are underpowered in these contexts. Moreover, meta-regression results are ecological and subject to confounding by study-level covariates, and they cannot establish causal relations at the individual level. Finally, survivorship bias and informative censoring may attenuate or accentuate long-term estimates depending on study-specific follow-up periods.

Publication-bias tests found no small-study effects and fail-safe N suggested robustness, but selective outcome reporting cannot be excluded. The predominance of data from developed countries may limit generalizability to healthcare systems with different resources and care models.

 These limitations warrant cautious interpretation and support for prioritizing individual-patient pooled analyses in future studies.

#### Clinical implications

These findings have several important clinical implications. First, the demonstration of persistent long-term mortality risk supports the need for extended medical surveillance of TBI survivors. Current practice models that focus primarily on acute and subacute care may inadequately address the chronic health risks identified in this analysis.

Second, the age-stratified results suggest that prognostic counseling should consider not only absolute mortality risk but also relative life expectancy reduction. Young TBI patients may benefit from particularly intensive long-term care strategies given their disproportionate relative mortality risk and longer potential years of life lost.

Third, the cause-specific mortality data indicate opportunities for targeted interventions. Suicide prevention protocols, cardiovascular risk management, infection control measures, and injury prevention strategies may be critical components of comprehensive TBI care. The elevated risk for external causes suggests that environmental modifications and safety counseling may be beneficial interventions.

Fourth, the temporal improvement in outcomes provides evidence that advances in TBI care can meaningfully impact long-term survival. Continued investment in research and quality improvement initiatives may yield further mortality reductions and improvements in quality of life for TBI survivors in future cohorts.

#### Gender-related differences in long-term outcomes

 Emerging evidence indicates that gender may influence long-term survival after TBI, with differences in hormonal regulation, neuroinflammatory responses, health-seeking behavior, and social reintegration potentially contributing to outcome variability. Women appear to exhibit distinct recovery trajectories and may experience different patterns of late mortality compared with men, although data remain limited and inconsistent across cohorts. Emerging evidence highlights the clinical importance of considering gender-related factors in both prognostic evaluation and rehabilitation planning [62]. Incorporating this perspective underscores the need for sex-disaggregated analyses in future research and supports the development of gender-sensitive longitudinal care strategies that can optimize outcomes for all TBI survivors.

#### Implications for healthcare systems

The substantial life expectancy reductions documented in this analysis represent significant economic and social burdens for healthcare systems and society. The average 7.6-year reduction in life expectancy translates to significant years of productive life lost, with implications for workforce participation, family dynamics, and healthcare resource utilization.

The persistent nature of mortality risk suggests that healthcare systems should develop chronic disease management models for TBI populations, like those established for other chronic conditions, such as diabetes or heart failure. These models may include regular medical surveillance, preventive interventions, care coordination, and patient education programs designed to address the multisystem risks identified in this analysis.

### Future research directions

Several research priorities emerge from these findings. First, targeted investigations into the underlying biological and physiological mechanisms, from cellular and molecular changes to alterations in brain networks, and informed by both basic science and clinical research, are needed to clarify the pathophysiological processes driving the persistent mortality risk after traumatic brain injury. Investigation of autonomic dysfunction (Paroxysmal Sympathetic Hyperactivity), neuroinflammation, hormonal alterations, and other potential mediators may identify therapeutic targets for intervention.

Second, treatment studies examining specific strategies to reduce long-term mortality risk are warranted. These might include cardiovascular risk modification programs, mental health interventions, comprehensive medical surveillance protocols, or novel neuroprotective strategies for the chronic phase of TBI recovery.

 Third, health services research examining optimal models of long-term TBI care could inform healthcare delivery strategies [63]. Comparative effectiveness studies of different care models, cost-effectiveness analyses, and implementation research may guide the development of sustainable long-term care programs.

Finally, prospective cohort studies with standardized outcome measures and extended follow-up periods would strengthen the evidence base and allow more precise risk stratification. Such studies should incorporate a detailed assessment of potential mediating factors and address the limitations of retrospective designs that characterize much of the existing literature.

## Conclusion

 This meta-analysis, which builds upon and extends our prior systematic review, demonstrates that TBI is associated with a significant reduction in life expectancy among severe TBI patients, particularly those with impaired functional independence (walking and feeding behavior) [31], as well as substantial and persistent elevation in mortality risk across all severity levels. The findings reveal a crucial role of age-related, time onset, and cause-specific mortality, which have important implications for clinical practice and healthcare policy. While outcomes have improved over time, significant challenges remain in optimizing long-term survival for TBI populations since they continue to face elevated mortality many years after injury. Even though the methodological heterogeneity and residual uncertainties detailed above suggest caution, these results support the necessity to develop comprehensive, longitudinal care models that address the multisystem risks faced by TBI survivors throughout their extended period of vulnerability.

## Supplementary Information

Below is the link to the electronic supplementary material.

Supplementary file 1
