# Supplementary material for: Severe traumatic brain injury and long-term survival: a meta-analysis on life expectancy and mortality trends
Source: Neurol Sci. 2026 Apr 10;47(5):409. doi: 10.1007/s10072-025-08706-6 (PMC13065621; doi:10.1007/s10072-025-08706-6)
Supplement: Supplementary file 1 [file 10072_2025_8706_MOESM1_ESM.docx]

**Supplementary 1. Search strategy**

**Pubmed**

(("brain injuries, traumatic"[MeSH Terms] OR "Trauma Severity Indices"[MeSH Terms] OR "Disability Evaluation"[MeSH Terms] OR "Severe Brain Injury"[Text Word] OR "Traumatic brain injury"[Text Word] OR "TBI"[Text Word] OR "severe TBI"[Text Word] OR "head trauma"[Text Word] OR "brain injury"[Title/Abstract] OR "head injury"[Title/Abstract] OR "traumatic brain"[Title/Abstract]) AND ("life expectancy/ethnology"[MeSH Terms] OR "life expectancy/history"[MeSH Terms] OR "life expectancy/trends"[MeSH Terms] OR "survival rate/trends"[MeSH Terms] OR "Healthy Life Expectancy"[MeSH Terms] OR "Life Expectancy"[MeSH Terms] OR "Longitudinal Studies"[MeSH Terms] OR "Risk Factors"[MeSH Terms] OR "Survival Rate"[MeSH Terms] OR "Long-Term Care"[MeSH Terms] OR "Life Expectancy"[Text Word] OR "Survival Rate"[Text Word] OR "standardized mortality ratio"[Text Word] OR "health expectancy"[Text Word] OR "long-term survival"[Text Word] OR "mortality"[Text Word] OR "survival"[Title/Abstract] OR "overall survival"[Title/Abstract] OR "years of life lost"[Title/Abstract] OR "YLL"[Title/Abstract] OR "SMR"[Title/Abstract]) AND ("italy"[MeSH Terms] OR "germany"[MeSH Terms] OR "United States"[MeSH Terms] OR "australia"[MeSH Terms] OR "europe"[MeSH Terms] OR "North America"[MeSH Terms] OR "oceania"[MeSH Terms]) AND "Humans"[MeSH Terms] AND 2000/01/01:2025/07/31[Date - Publication]) NOT ("Animals"[MeSH Terms] NOT "Humans"[MeSH Terms])

Results July 2025: 9,767 articles

**Cochrane Library**

("Brain Injuries, Traumatic" OR "Trauma Severity Indices" OR "Disability Evaluation"

OR "Severe Brain Injury" OR "Traumatic brain injury" OR TBI OR "severe TBI" OR "head trauma"

OR "brain injury" OR "head injury" OR "traumatic brain")

AND

("Life Expectancy" OR "Survival Rate" OR "Healthy Life Expectancy" OR "Longitudinal Studies"

OR "Risk Factors" OR "Long-Term Care" OR "life expectancy" OR "survival rate" OR "standardized mortality ratio"

OR "health expectancy" OR "long-term survival" OR mortality OR survival OR "overall survival"

OR "years of life lost" OR YLL OR SMR)

AND

(Italy OR Germany OR "United States" OR Australia OR Europe OR "North America" OR Oceania)
Results July 2025: 8 Cochrane Reviews; 308 trials

**Google Scholar**

intitle:"traumatic brain injury" OR intitle:"severe TBI"

AND ("long-term survival" OR "overall survival" OR "standardized mortality ratio" OR SMR OR YLL)

AND (Italy OR Germany OR "United States")

Results July 2025: 825 articles

**PEDro**
(severe traumatic brain injury OR severe TBI OR head trauma OR brain injury OR traumatic brain)

AND (life expectancy OR survival OR mortality OR "survival rate" OR "long-term survival" OR "standardized mortality ratio")
Results July 2025: No records found

**EMBASE**

('brain injury'/exp OR 'traumatic brain injury'/exp OR 'severe traumatic brain injury' OR 'severe TBI' OR 'head trauma'

OR 'brain injury':ti,ab OR 'head injury':ti,ab OR 'traumatic brain':ti,ab)

AND

('life expectancy'/exp OR 'survival rate'/exp OR 'healthy life expectancy'/exp OR 'longitudinal study'/exp

OR 'risk factor'/exp OR 'long term care'/exp OR 'life expectancy':ti,ab OR 'survival rate':ti,ab

OR 'standardized mortality ratio':ti,ab OR 'health expectancy':ti,ab OR 'long-term survival':ti,ab

OR mortality:ti,ab OR survival:ti,ab OR 'overall survival':ti,ab OR 'years of life lost':ti,ab OR YLL:ti,ab OR SMR:ti,ab)

AND

('italy'/exp OR 'germany'/exp OR 'united states'/exp OR 'australia'/exp OR 'europe'/exp OR 'north america'/exp OR 'oceania'/exp)

AND [2000-2025]/py

AND [humans]/lim

Results July 2025: 5,367

**Supplementary 2. Risk bias assessment**

We retained the risk of bias assessment tools used in the previous review^17^ to ensure methodological consistency and to extend the quality appraisal to any new studies identified during the additional search period. The Methodological Index for Non-Randomized Studies (MINORS) was used to evaluate key methodological aspects of non-randomized designs, including clarity of aims, adequacy of patient selection, data collection methods, endpoint relevance, follow-up completeness, and appropriateness of statistical analyses. The Oxford Centre for Evidence-Based Medicine (OCEBM) 2011 Levels of Evidence provided a hierarchical classification of study design and rigor. The Newcastle-Ottawa Scale (NOS) was additionally adopted to assess cohort and case–control studies across three domains: selection (up to four stars), comparability (up to two stars), and outcome or exposure ascertainment (up to three stars), for a total of nine stars. Studies scoring ≥7 were rated as high quality with low risk of bias, 4–6 as moderate quality, and <4 as low quality.

**Supplementary Methods: Statistical Procedures**

Standardized mortality ratios (SMRs) were extracted or computed by comparing the observed number of deaths in TBI populations with the expected number based on age-, sex-, and calendar-year–adjusted mortality rates in the general population. Hazard ratios (HRs) were included when reported or derived from Kaplan–Meier survival curves using validated reconstruction techniques. Cumulative survival proportions at predefined time points (e.g., 1, 5, and 10 years) were recorded where available. Dichotomous outcomes (e.g., all-cause mortality) were expressed as risk ratios (RRs) or odds ratios (ORs), while continuous outcomes (e.g., mean survival time or years of life lost, YLL) were summarized using mean differences (MDs) with 95% confidence intervals (CIs).

In addition to 95% CIs, 95% prediction intervals (PIs)³¹^,^³² were calculated to reflect the expected range of effects in future comparable populations. Between-study heterogeneity was assessed using Cochran’s Q test and quantified by the I² statistic, with values of 25%, 50%, and 75% representing low, moderate, and high heterogeneity, respectively³³^,^³⁴. The between-study variance (τ²) was estimated using the restricted maximum likelihood (REML) method²⁷,²⁸ and reported for all random-effects models.

Meta-regression analysis were performed to explore the influence of continuous study-level covariates, including study midpoint (calendar year), mean participant age, proportion of severe TBI cases, follow-up duration (years), and total sample size (log-transformed).³⁵^,^³⁶ Robust standard errors were estimated using the Hartung–Knapp adjustment.³⁶ The proportion of explained heterogeneity (pseudo-R²) and residual τ² were reported as measures of model fit, and variance inflation factors (VIFs) were computed to assess multicollinearity. For life expectancy outcomes, weighted mean differences in YLL were pooled, with stratified analyses by injury severity.

To evaluate the robustness and generalizability of the findings, the dataset was subsequently expanded to include up to fourteen eligible studies, incorporating those without directly reported SMRs and 95% confidence intervals. For these studies, SMRs were reconstructed from alternative effect measures, including hazard ratios, mortality rate ratios, or survival probabilities derived from published Kaplan–Meier curves, using standardized reconstruction methods as described in previous methodological literature.

Sensitivity analyses included fixed-effects modeling and leave-one-out diagnostics to test robustness³⁷. Influence analyses (including Baujat plots)³⁸ were used to identify studies contributing disproportionately to heterogeneity or pooled estimates. Publication bias was assessed through visual inspection of funnel plots, Egger’s regression asymmetry test³⁹, Begg’s rank correlation test⁴⁰, and the trim-and-fill procedure when ≥10 studies were available⁴¹. Classic fail-safe N calculations⁴² were also performed to estimate the number of missing studies required to nullify the observed effect.

**Supplementary 3. Detailed Quality Assessment Results**

The mean MINORS score was 15.8 (range, 14–18 out of 18), with a median of 16. Nineteen studies (76%) scored 16, one study scored 18 (4%), three studies scored 15 (12%), and two studies scored 14 (8%). Overall, most studies scored ≥16, indicating good methodological quality with clearly stated aims, inclusion of consecutive participants, appropriate endpoints, adequate follow-up, and sound statistical analyses.

The NOS scale was applied to all eligible cohort studies; one study was excluded from this assessment due to its cross-sectional design⁵⁹. The median overall score was 7 (range, 6–8 out of 9), with sixteen studies meeting the high-quality threshold (≥7 stars). Most studies achieved full scores in the selection and outcome domains, while comparability between groups was more variably addressed.

**Supplementary 4. Age-Related Mortality Patterns in the Recent Era (2004–2019)**

| **Age Group (years)** | **No. of Studies** | **Participants** | **SMR** | **95% CI** | **I² (%)** | **τ²** | **Prediction Interval** | **Odds Ratios (vs ≥75 years)** |
| --- | --- | --- | --- | --- | --- | --- | --- | --- |
| 15–34 | 4 | 38,891 | 6.23 | 4.12 – 9.41 | 31.2 | 0.107 | 3.45 – 11.25 | 2.58 (95% CI 1.67–3.99,  p < 0.001) |
| 35–54 | 4 |  | 7.18 | 5.34 – 9.65 | 28.9 | 0.091 | 4.12 – 12.51 | 2.98 (95% CI 2.01–4.42,  p < 0.001) |
| 55–74 | 4 |  | 3.94 | 3.11 – 4.99 | 22.1 | 0.052 | 2.78 – 5.59 | Reference |
| ≥75 | 4 |  | 2.41 | 1.98 – 2.94 | 19.8 | 0.035 | 1.72 – 3.37 | Reference |

**Supplementary 5. Temporal Patterns of Mortality Risk by Follow-Up Duration and Injury Severity**

| **Injury Severity** | **Follow-up Period** | **No. of Studies** | **SMR** | **95% CI** | **I² (%)** | **τ²** | **Prediction Interval** | **Trend Test (z, p)** |
| --- | --- | --- | --- | --- | --- | --- | --- | --- |
| Severe TBI | < 2 years | 6 | 6.34 | 5.21 – 7.72 | 15.2 | 0.032 | 4.52 – 8.90 | z = −4.21, p < 0.001 |
|  | 2–5 years | 6 | 4.18 | 3.47 – 5.03 | 28.7 | 0.046 | 2.90 – 6.03 |  |
|  | 5–10 years | 6 | 3.21 | 2.67 – 3.86 | 31.4 | 0.051 | 2.10 – 4.91 |  |
|  | > 10 years | 6 | 2.87 | 2.31 – 3.57 | 34.2 | 0.054 | 1.90 – 4.32 |  |
| Moderate TBI | < 2 years | 4 | 3.89 | 2.94 – 5.14 | 22.1 | 0.039 | 2.30 – 6.59 | z = −2.89, p = 0.004 |
|  | 2–5 years | 4 | 2.67 | 2.12 – 3.36 | 18.9 | 0.031 | 1.75 – 4.07 |  |
|  | ≥ 5 years | 4 | 2.14 | 1.78 – 2.57 | 24.3 | 0.042 | 1.42 – 3.22 |  |
